# Radiofrequency-Wire Assisted Recanalization of Chronically Occluded Iliofemoral Venous Stents

**DOI:** 10.1055/a-2804-6030

**Published:** 2026-02-27

**Authors:** Ernest Barral, Kush R. Desai

**Affiliations:** 1 Department of Radiology, Northwestern University Feinberg School of Medicine, Chicago, Illinois, United States; 2 Division of Vascular and Interventional Radiology, Department of Radiology, Northwestern University Feinberg School of Medicine, Chicago, Illinois, United States

## Introduction

Chronic venous occlusions (CVOs) occur when veins become obstructed due to thrombus formation, with subsequent organization into an occlusion by collagenous material. 1 2 Iliofemoral venous obstruction is a well-recognized cause of lower extremity venous hypertension and may manifest as limb swelling, pain, venous claudication, skin changes, or ulceration in advanced disease. 1 3 Endovascular therapy has become the primary treatment strategy for symptomatic iliofemoral venous obstruction, with the goal of restoring venous outflow and improving quality of life. In many cases, this requires venous stent placement to address fixed anatomic obstruction and maintain long-term patency. Despite technical advances, venous stents are vulnerable to failure over time, most commonly due to in-stent restenosis (ISR) or stent occlusion.

The primary patency rates of venous stents depend on multiple factors, including the inflow-outflow veins, adherence to anticoagulation therapy, and mechanical failure of the stent. 4 Maintaining long-term stent patency remains a significant challenge, as patency rates decline within 4 to 5 years in lower extremity vessels, often resulting in recurrence of symptoms and reintervention. ISR, stent compression, or total occlusion of the stents is associated with recurrence of the symptoms. 4 Restenosis and occlusion of venous stents are primarily characterized by thrombosis and subsequent organization into type 1 and 3 collagen. 2 5 6 7 8 Stents placed for postthrombotic obstructions have primary patencies reported as 88% at 12 months, and 81.6% at 36 months. 9 10 Projected 5-year primary patency in chronic postthrombotic obstructions is approximately 60%. 11

Crossing stent occlusions represents a rate-limiting step in endovascular intervention. Initial attempts to recanalize venous stent occlusions involve conventional tools, for example, hydrophilic guidewires advanced through well-supported coaxial or triaxial catheter systems to penetrate the occlusion. 3 The success of conventional tools relies on the physical characteristics of the occlusion. The composition of chronic stent occlusions is variable and classified into two types: soft lesions and hard lesions. 6 12 13 Softer lesions are often thrombotic in nature, whereas more resistant hard lesions consist of organized thrombus with dense collagen deposition, fibrosis, or calcification. These rigid, hard occlusions require substantially greater penetration force than soft thrombotic lesions and frequently challenge conventional guidewire techniques. Failure to cross may result from wire buckling, loss of directional control, or deviation from the intended intraluminal course. 3 When conventional tools fail to cross occlusions, several sharp needle-based crossing tools can be deployed. 1 14 15 16 17 18 19 However, there remains a subset of cases with heavily fibrotic occlusions that are resistant to both conventional and sharp recanalization tools and require alternative tools for crossing the occlusion.

The PowerWire (Baylis Medical Technologies, Toronto, Canada) radiofrequency (RF) guidewire-assisted recanalization has shown high success rates in crossing occlusions in both native and stented vessels. 3 13 20 21 22 23 24 25 26 RF-assisted recanalization has been applied across a range of complex refractory vascular occlusions, including peripheral arterial chronic total occlusions, central venous occlusions, and advanced iliocaval disease. This technology enables controlled delivery of RF energy at the wire tip, facilitating advancement through dense fibrotic lesions with less mechanical force than required for conventional guidewires. 27 By reducing the reliance on forward pressure alone, the RF-wire assisted technique may mitigate failure to cross due to buckling or uncontrolled deflection. Additionally, an advantage of RF-wire during stent recanalization is its response to metal contact. 3 Upon contact with the stent material, the system automatically interrupts RF energy delivery, limiting further advancement and potentially reducing the risk of unintended passage beyond the stent lumen. This attribute may be particularly useful when navigating long or complex stent occlusions, where maintaining a central intraluminal trajectory is critical.

**Fig. 1:**
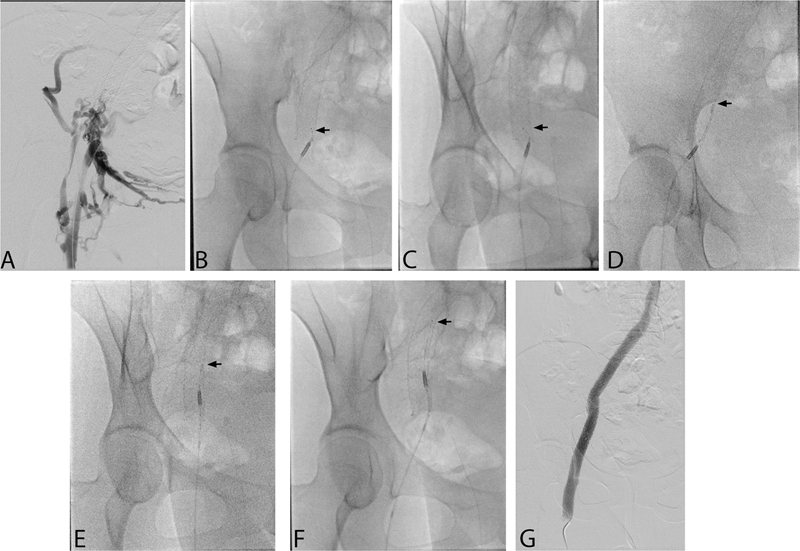
Case 1: 33-year-old female with a history of left lower extremity deep vein thrombosis and development of postthrombotic syndrome with occlusion of previously placed self-expanding left iliac venous stent. ( **A** ) Preprocedural venogram demonstrating severe (greater than 75%) stenosis of the left common femoral vein and postthrombotic occlusion of the left common/external iliac vein stent. ( **B–F** ) Multiple fluoroscopic obliquities were obtained while utilizing the 110-g PowerWire RF 40-degree angled wire (tip marked with a black arrow) with a TriForce crossing system (Cook Medical, Bloomington, Indiana, United States) to ensure intraluminal trajectory while incrementally traversing the stent occlusion. ( **G** ) Completion venogram demonstrates subtotal luminal restoration following additional left iliac and femoral stent placement.

The purpose of this article is to describe our experience in utilizing RF wire to cross stent occlusions within the iliofemoral venous system. We present a series of cases ( Figs. 1 2 3 4 ) where PowerWire RF wire was successfully used to cross complex stent occlusions. We also report a case in which a chronically occluded left common/external iliac stent appears to have eroded into the adjacent extravascular space, no longer sharing the common channel with the native common iliac vein and infrarenal IVC ( Fig. 4 ). This case emphasizes the importance of comprehensive preprocedural planning using cross-sectional imaging to accurately delineate vascular anatomy and identify adjacent structures at risk for injury. Furthermore, it highlights the need for employing multiple intraprocedural fluoroscopic obliquities and meticulous triangulation to ensure an appropriate intraluminal trajectory is maintained.

**Fig. 2:**
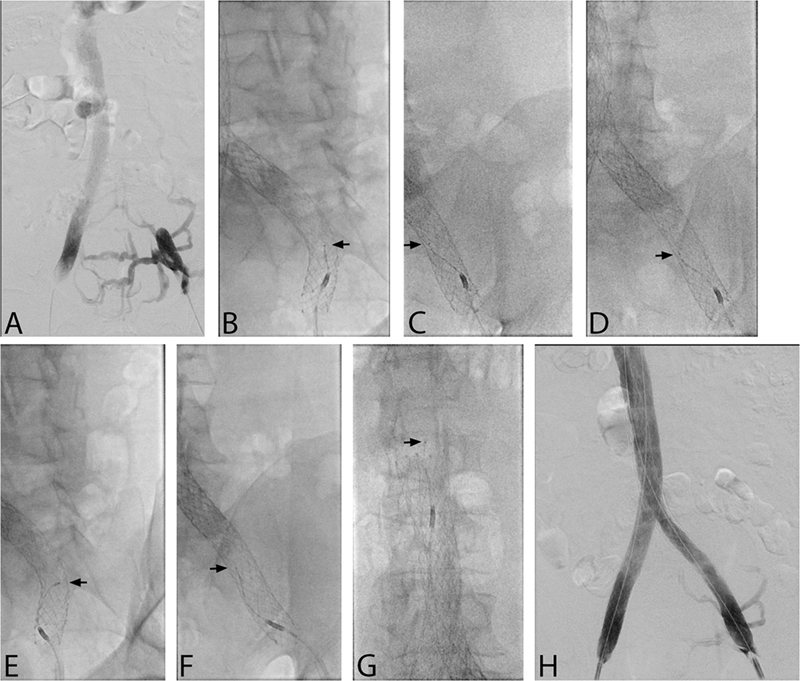
Case 2: 56-year-old male with significant venous thromboembolism history complicated by difficulties with anticoagulation, with chronic IVC thrombosis and occlusion of left external/common iliac/IVC stent moiety who presented with recurrent left leg swelling and discomfort. ( **A** ) Preprocedural venography was performed, demonstrating occlusion of the left external iliac/left common iliac/left inferior vena cava stent moiety with a patent left common femoral vein draining into pelvic collaterals. Initial attempts to cross the occlusion in the left external/common iliac vein were performed utilizing the angled tip catheter and stiff hydrophilic wire (Glidewire, Terumo, Somerset, New Jersey, United States), without success. ( **B–G** ) Multiple fluoroscopic obliquities were obtained while utilizing the 110-g PowerWire RF 40-degree angled wire (tip marked with a black arrow) with a TriForce crossing system to ensure intraluminal trajectory while incrementally traversing the stent occlusion. ( **H** ) Completion venography and IVUS were performed, demonstrating subtotal luminal restoration and cessation of collateral opacification.

**Fig. 3:**
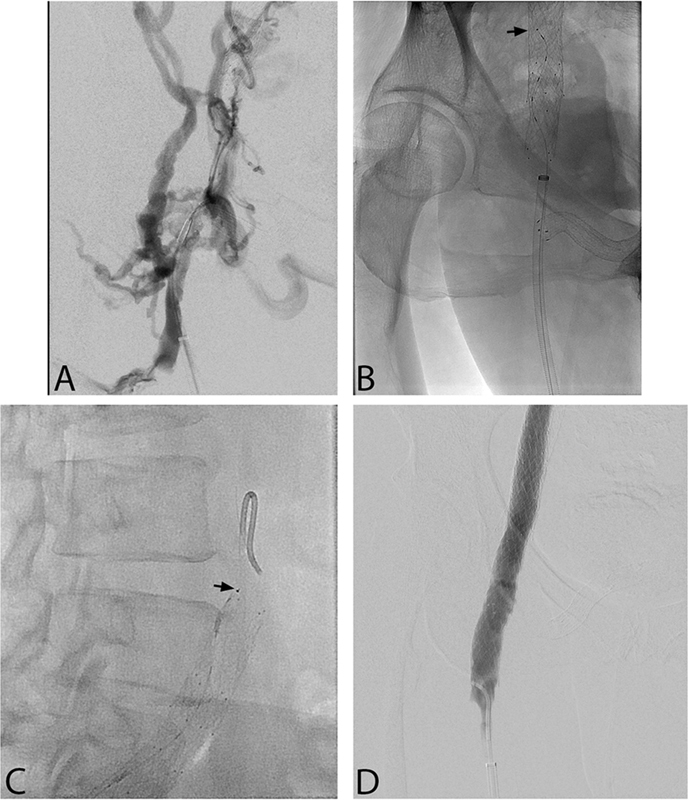
Case 3: 55-year-old female with significant left lower extremity postthrombotic syndrome on the basis of an occluded left iliac vein stent. ( **A** ) Selective venography of the left common femoral vein demonstrated occlusion of the mid left common femoral vein through the left common/external iliac vein stents in place. Collateral drainage is present via pelvic collaterals. ( **B, C** ) Using a combination of stiff Glidewire and 110-g PowerWire RF 40-degree angled wire (tip marked with a black arrow), the existing occluded left iliofemoral stent construct was traversed. Contralateral venous access was used as well, with a reverse curve catheter serving as a target to facilitate successful crossing. ( **D** ) Completion venograms were obtained following stent placement and balloon dilation, demonstrating subtotal luminal restoration.

**Fig. 4:**
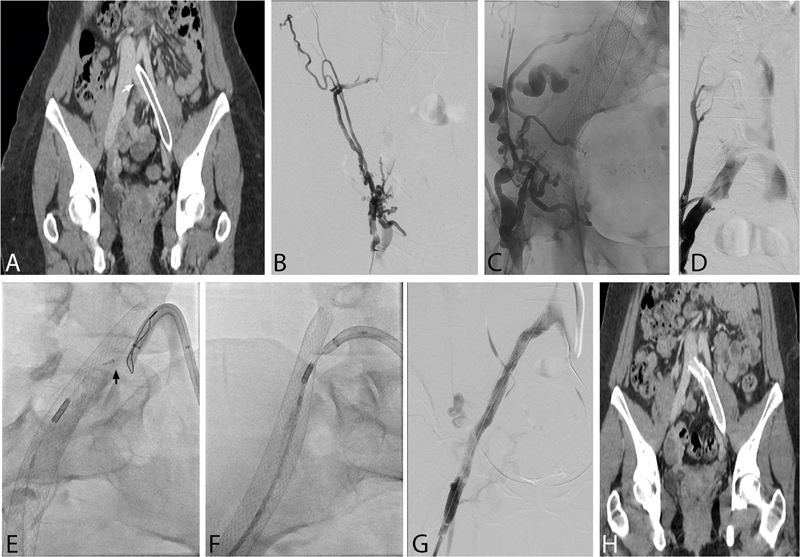
Case 4: 37-year-old female presenting with a history of May–Thurner syndrome and chronically thrombosed left iliac vein stent. Prior cross-sectional imaging demonstrated that the lateral margin of the stent had eroded through the iliac vein wall and was likely extravascular. ( **A** ) Computed tomography venogram shows the chronically occluded left iliac stent adjacent to a patent cranial portion of the left iliac vein, demonstrating that the lateral margin of the stent has eroded through the cranial margin of the left iliac vein and is partially extravascular (marked with white arrow). ( **B, C** ) Left femoral venograms demonstrating chronic postthrombotic occlusion of the left common and external iliac veins. Initial attempts to access the caudal portion of the occluded stent were initially unsuccessful. ( **D** ) Selective venography of the cranial iliac venous segments was performed, confirming a patent portion of the cranial left common iliac vein and demonstrating fluoroscopically that the occluded left iliac venous stent had eroded through the cranial margin of the left iliac vein. ( **E, F** ) Spot radiograph demonstrating a 15 mm gooseneck snare (Amplatz, Medtronic, Minneapolis, Minnesota, United States), placed from the contralateral right popliteal access site into the patent portion of the proximal left iliac vein via a direction guide sheath (Agilis, Abbott, Abbott Park, Illinois, United States) adjacent to the cranial aspect of the occluded stent. With care to avoid the anatomic location of the adjacent iliac arteries, the 110-g PowerWire RF 40-degree angled wire (tip marked with a black arrow) was then used to carefully exit the occluded stent through an interstice and into the gooseneck snare to achieve through-and-through access. ( **G** ) Completion venogram following additional venous stent placement into the left common/external iliac vein and left common femoral vein demonstrates subtotal luminal restoration. ( **H** ) Postprocedural computed tomography demonstrates that the new left iliac and common femoral vein stents are widely patent. An excluded extravascular portion of the original chronically occluded left iliac venous stent is partially visualized adjacent to the patent proximal left iliac stent.

## Discussion

RF wire use has been shown to be effective in native and stented vessel occlusions. Previous studies have reported the use of PowerWire in crossing long native occlusions exceeding 3 cm. 20 21 22 24 28 RF wire use in long native venous occlusions requires careful technique to prevent the risk of forming a new tract rather than recanalizing the existing lumen, thereby increasing the likelihood of deviation into adjacent structures. In our experience, RF wire is generally most valuable in chronically occluded stented segments, where the stent provides a defined anatomical target and minimizes the risk of creating an unintended tract. This technology can also be advantageous in select short native venous occlusions, typically those measuring approximately 1 cm or less, where the operator can reasonably assume close proximity between the proximal and distal true lumens. In such cases, the risk of injury to adjacent structures is comparatively lower, particularly in large-caliber vessels.

Our preferred technique involves advancing a 40-degree angled RF wire through a dedicated crossing catheter, such as the TriForce catheter. This configuration enables controlled directionality and incremental advancement across the occlusion. Throughout the procedure, confirmation of position within the stent must be done in multiple obliquities; wire passage is always done under live fluoroscopy. Occasionally, small-diameter balloon angioplasty may need to be performed to allow for adequate positioning within the stent and to alter the trajectory of wire passage. Once substantial progress has been achieved and intraluminal access is suspected or confirmed, the RF wire is typically exchanged for a 0.035-inch stiff Glidewire to complete the crossing and facilitate subsequent interventions.

Although RF wire technology offers a safety advantage when used in crossing stent occlusions, its use is not without inherent risk. The wire may traverse the gaps between stent struts (interstices), resulting in extraluminal advancement. Consequently, RF wire application necessitates meticulous technique, continuous monitoring of wire position, and thoughtful case selection. Operators must possess a thorough understanding of adjacent critical structures and the potential consequences of wire deviation. Risk assessment should be conducted before RF activation, and wire advancement should be deliberate, with frequent reassessment. The overarching goal is to minimize complications through meticulous technique combined with judicious patient and lesion selection.

In summary, RF wire technology represents a valuable adjunct for the recanalization of chronically occluded venous stents when used in appropriate clinical scenarios. Its ability to interact with metallic stents (metal contact error) can facilitate intraluminal crossing; however, it does not eliminate the need for meticulous technique and precise anatomical awareness. Achieving optimal outcomes depends on careful case selection, controlled wire advancement, and a clear understanding of both the advantages and limitations of RF wire use.
